# Diagnostic Accuracy of Ultrasound in Predicting Extrathyroidal Extension and Its Relation to Body Mass Index in a North American Population

**DOI:** 10.3390/biomedicines10102408

**Published:** 2022-09-26

**Authors:** Mahmoud Omar, Abdallah S. Attia, Peter P. Issa, Bryce R. Christensen, Kavin Sugumar, Ahmed Alnahla, Deena Hadedeya, Hosam Shalaby, Neel Gupta, Mohamed Shama, Eman Toraih, Emad Kandil

**Affiliations:** 1 Department of Surgery, School of Medicine, Tulane University, New Orleans, LA 70112, USA; 2 School of Medicine, Louisiana State University Health Sciences Center, New Orleans, LA 70112, USA; 3 Department of Radiology, School of Medicine, Tulane University, New Orleans, LA 70112, USA; 4 Genetics Unit, Department of Histology and Cell Biology, Faculty of Medicine, Suez Canal University, Ismailia 41522, Egypt

**Keywords:** papillary thyroid cancer, ultrasound, extrathyroidal extension, ETE, body mass index

## Abstract

Detection of extrathyroidal extension (ETE) in patients with papillary thyroid carcinoma (PTC) influences treatment plan and surgical aggressiveness. Ultrasound (US) is the long-standing preoperative imaging method of choice. Recent literature from Asia suggests US accuracy to be influenced by patient characteristics, such as body mass index (BMI). Here, we examine the effect of BMI on the accuracy of US at a North American tertiary referral center. A total of 204 PTC-confirmed patients were retrospectively read by a radiologist blinded to surgical pathology findings. The radiologist recorded multiple sonographic features, including ETE, loss of echogenic capsule, nodule vascularity, capsular abutment, and bulging of contour. When considering all patients, the ultrasonographic feature with the best overall performance was loss of echogenic capsule (diagnostic odds ratio (DOR) = 4.48, 95% confidence interval (CI) = 1.86–10.78). Sub-group analysis by patient BMI found that area under the curve (AUC) for sonographic features was greater in non-obese BMI patients (0.71 ± 0.06) when compared with obese patients (0.43 ± 0.05; *p* = 0.001). Overall, US diagnostic performance was significantly better in non-obese (DOR = 3.70, 95%CI = 1.53–8.94) patients when compared to those who were obese (DOR = 1.12, 95%CI = 0.62–2.03; *p* = 0.03). Loss of the echogenic capsule did not differ between the two cohorts with respect to DOR (*p* = 0.51), specificity (*p* = 0.52), or sensitivity (*p* = 0.09). Our work suggests that the diagnostic value of ETE detection by US is impaired in obese patients. Considering that loss of the echogenic capsule did not differ with respect to diagnostic performance, specificity, nor sensitivity between non-obese and obese patients, it could be considered the most important predictor of US-determined ETE.

## 1. Introduction

Thyroid cancer is the fastest growing cancer in the United States, with papillary thyroid carcinoma (PTC) being the most common endocrine malignancy [1]. PTC generally has excellent prognosis, especially if it is confined to the thyroid gland [2,3]. Still, studies have identified risk factors of disease progression in PTC patients [4,5,6]. Extrathyroidal extension (ETE), or extension of the cancer beyond the thyroid capsule itself, is a significant predictive factor of poor prognosis, influencing both disease recurrence and survival [4,7,8,9,10,11]. While most of all patients with PTCs confined to the thyroid gland can expect to survive past 20 years [12], patients with PTC and ETE suffer comparatively worse 15–20 year survival rates [12,13]. Therefore, accurate ETE evaluation and determination may influence the recommended surgical extent and the need for adjuvant therapy [11].

Considering the difficulty of accurate detection on preoperative and intraoperative assessment, ETE detection continues to be largely determined on postoperative pathological examination [2]. Imaging modalities to detect ETE preoperatively include ultrasound (US), computed tomography (CT), and magnetic resonance imaging (MRI). Of these imaging modalities, US is the most frequently utilized. US determination of ETE has been shown to be superior to other imaging modalities with an accuracy as high as 83.3% [14].

Despite the efficacy of ETE determination, US findings fluctuate with respect to specific parameters. For example, transducer frequency and operator/user experience are two factors which impact the accuracy of detection by US [15,16]. Importantly, body mass index (BMI) influences the accuracy of US diagnosis, as image resolution decreases with increasing depth of insonation [17]. In relation to the thyroid, the distance-dependent nature of ultrasound makes accurate evaluation more challenging with increasing levels of adipose tissue between the receiver and target of study [18]. One study analyzing 625 patients with PTC found poorer negative predictive values (50% vs. 72.6%, *p* = 0.010) and accuracy (53.1% vs. 71.7%, *p* = 0.025) in detecting lymph node metastasis on US in obese (≥30 kg/m^2^) versus non-obese (<30 kg/m^2^) patients [19]. 

Recently, Kamaya et al. described diagnostic features that are important in evaluating ETE in patients with thyroid cancer, including capsular abutment, contour bulging, vascularity beyond the capsule, as well as loss of the echogenic capsule [2]. Considering the importance of ETE determination on PTC management, we investigated ultrasonographic features predictive of ETE, with specific interest in the influence of patient BMI. 

## 2. Methods

A single-institution retrospective study was performed after obtaining institutional review board (IRB) approval. Patients diagnosed with PTC who underwent surgical treatment were included from our prospectively collected database. Only patients with PTC determined by surgical pathology were included. All patients underwent thyroidectomy (hemithyroidectomy or total thyroidectomy) by the same high-volume fellowship-trained endocrine surgeon (E.K.) with more than 15 years of experience. 

Patient demographics, including age, gender, and BMI, as well as surgical pathology reports were collected. All patients completed a preoperative comprehensive neck US evaluation using a high-resolution US system (GE Logiq 9) equipped with a high-frequency linear-array transducer (14 MHz) for thyroid gland evaluation. Patients without a complete comprehensive neck US assessment were excluded from the study. US images were retrospectively reviewed by an expert author in thyroid imaging who was blinded to the original ultrasound report itself as well as the final pathology report at the time of evaluation (N.G., radiologist). Assessment of ETE on US imaging was determined by evaluating capsular abutment, bulging of thyroid contour, loss of the echogenic capsule, and vascularity extending beyond the nodule. Capsular abutment was identified if the thyroid nodule was seen abutting the thyroid capsule. Contour bulging was identified if the anterior capsule was seen bulging outwards by the nodule. Loss of the echogenic capsule was determined if the echogenic rim of the thyroid gland was not seen on ultrasound. Extension of vascularity beyond the nodule was identified if vessels extending to or from the nodule were seen beyond the capsule on power Doppler. Subjective assessment of ETE on US was also determined. Each ultrasonographic feature was evaluated as either present or absent. Primary analysis determined the diagnostic accuracy with which ultrasonographic features could accurately determine ETE. Subsequent analysis stratified patients into two groups by patient BMI, including non-obese (<30 kg/m^2^) and obese (≥30 kg/m^2^) cohorts, to determine the effect of US accuracy with relation to BMI, if any. 

For statistical analysis, a Pearson chi-square or Fisher’s exact test were used as appropriate for categorical variables. To compare the groups and their mean values, sample *t*-tests or Wilcoxon rank sum tests were used. Normality distribution was tested by a Shapiro–Wilk test. Statistical significance was set at a two-tailed *p*-value of <0.05. Analyses were performed with SAS 9.4 (SAS Institute Inc., Cary, NC, USA). Diagnostic accuracy was assessed for each sonographic feature using Meta-DiSc software (version 1.4). The accuracy of the diagnostic test was presented in terms of sensitivity, specificity, as well as positive and negative likelihood ratios. Confidence intervals of 95% (95%CI) were Wilson-score derived for sensitivity and specificity. The diagnostic odds ratio (DOR) was calculated. The 95%CI for DOR and likelihood ratios were Wald-type derived. A summary receiver operator characteristics curve (ROC) was constructed to assess parameter accuracy. 

## 3. Results

A total of 204 PTC patients were included, of which 49 (24.0%) had pathology-proven ETE (Table 1). Patients with ETE and without ETE were similar with respect to age (49.4 ± 15.8 versus 50.4 ± 15.2 years, respectively, *p* = 0.71) and sex (93% versus 82% female, respectively, *p* = 0.14). The BMI of patients with ETE was 31.1 ± 8.4 while the BMI of those without ETE was 32.0 ± 8.9 kg/m^2^ (*p* = 0.24). There were 6 patients (2.94%) in the non-obese cohort and 36 patients (17.64%) in the obese cohort with ETE (*p* = 0.35). 

**Figure 1:**
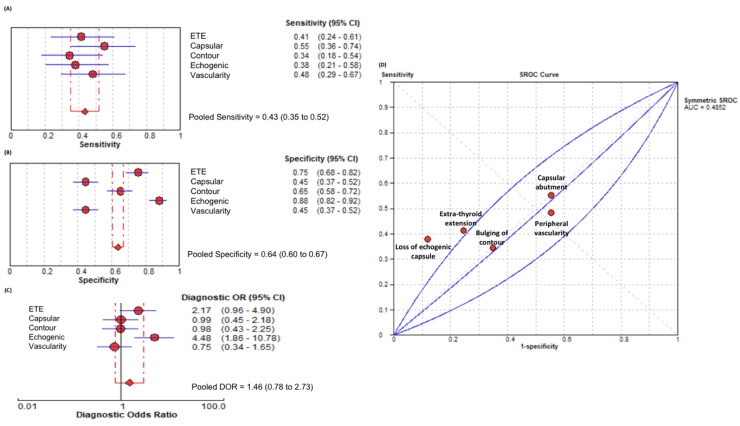
Pooled analysis of different sonographic features. The (**A**) sensitivity, (**B**) specificity, (**C**) diagnostic odds ratio, and (**D**) summary receiver operating curve are depicted. ETE: subjective suspicion of ETE, Capsular: abutment of the capsule, Contour: bulging of thyroid contour, Echogenic: loss of echogenic capsule, Vascularity: extension of vascularity beyond thyroid capsule. CI: confidence interval; DOR: diagnostic odds ratio; sROC: summary receiver operating curve; AUC: area under the curve.

Mean tumor size was larger in patients with ETE (1.8 ± 1.4 cm) compared to those without ETE (1.2 ± 1.3 cm; *p* = 0.04). Multifocality was noted in 7.84% (*n* = 16) of patients with ETE and in 33.33% (*n* = 68) of patients without ETE (*p* = 0.09). Patient baseline characteristics are summarized in Table 1. The pooled sensitivity and specificity of ultrasound were 43% (95% confidence interval (CI) = 35–52%) and 64% (95%CI = 60–67%), respectively (Figure 1). 

The pooled DOR of ETE by US was 1.46 (95%CI = 0.78–2.73). Of the sonographic features, loss of echogenic capsule had the best diagnostic performance (DOR= 4.48, 95%CI = 1.86–10.78) with 88% specificity (95%CI = 82–92%) and 38% sensitivity (95%CI = 21–58%). Subjective assessment of ETE had the second highest diagnostic performance (DOR = 2.17, 95%CI = 0.96–4.90) with 75% specificity (95%CI = 68–82%) and 41% sensitivity (95%CI = 24–61%). The presence of capsular abutment, bulging of the contour, and extension of vascularity beyond the capsule were of poor diagnostic value. The presence of capsular abutment had a DOR of 0.99 (95%CI = 0.45–2.18) with 45% specificity (95%CI = 37–52%) and 55% sensitivity (95%CI = 36–74%). Bulging of the contour had a low diagnostic performance (DOR= 0.98, 95%CI= 0.43–2.25) with a specificity of 65% (95%CI = 58–72%) and sensitivity of 34% (95%CI = 18–54%). Finally, the DOR for the extension of vascularity beyond the capsule was non-informative (DOR = 0.75, 95%CI = 0.34–1.65) with a 45% specificity (95%CI = 37–52%) and 48% sensitivity (95%CI = 29–67%). 

**Figure 2:**
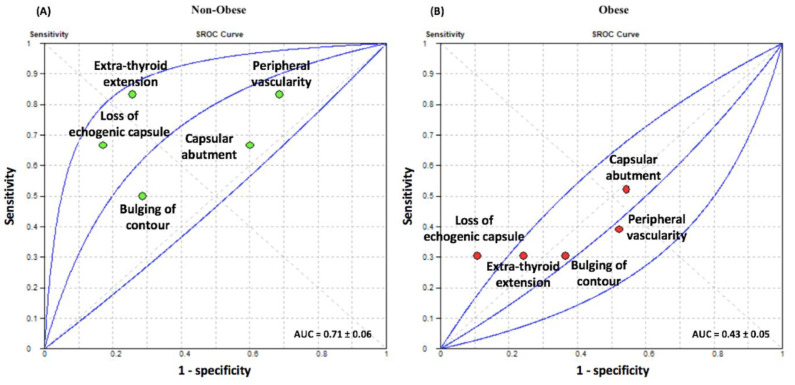
Receiver operating curve (ROC) of sonographic features in assessing for ETE. The summary receiver operating curve for (**A**) non-obese and (**B**) obese patients are depicted. Area under the curve is reported with corresponding a standard error. ETE: subjective suspicion of ETE, Capsular: abutment of the capsule, Contour: bulging of thyroid contour, Echogenic: loss of echogenic capsule, Vascularity: extension of vascularity beyond thyroid capsule. sROC: summary receiver operating curve; AUC: area under the curve.

After stratification by BMI, US predictive features, in general, were more accurate in non-obese patients when compared to those of obese patients (Table 2). The mean BMI in the obese cohort was 38.65 ± 7.28 kg/m^2^ and the mean BMI in the non-obese cohort was 24.96 ± 3.29 kg/m^2^. The area under the curve (AUC) for sonographic features in non-obese patients (0.71 ± 0.06) was significantly greater than that for obese patients (0.43 ± 0.05; *p* = 0.001), as shown in Figure 2. Though diagnostic performance of individual US features did not differ between the two cohorts, the overall diagnostic performance of US was significantly better in non-obese (DOR = 3.70, 95%CI = 1.53–8.94) patients when compared with those who were obese (DOR = 1.12, 95%CI = 0.62–2.03; *p* = 0.03). The pooled sensitivity of US differed between the two groups, with 70% (95%CI = 51–85%) and 37% (95%CI = 28–46%) sensitivities in non-obese and obese patients, respectively (*p* = 0.001). The specificity of ETE detection by US did not differ between the two groups with respect to individual US parameter nor when pooled (non-obese: 60%, 95%CI = 52–67%; obese: 64%, 95%CI = 61–68%; *p* = 0.32). The diagnostic odds ratio for loss of echogenic capsule in non-obese patients was 9.67 (95%CI = 1.43–65.38) and 3.65 (95%CI = 1.29–10.29) in obese patients (*p* = 0.51), with similar specificities (non-obese: 83%, 95%CI=66–93%; obese: 89%, 95%CI = 83–94%; *p* = 0.52). The non-obese cohort tended to have higher sensitivity (non-obese: 67%, 95%CI =22–96%; obese: 30%, 95%CI=13–53%) with respect to loss of echogenic capsule, though this difference was not significant (*p* = 0.09). Subjective assessment of ETE in the non-obese cohort had a significantly higher sensitivity (non-obese: 83%, 95%CI = 36–100%; obese: 30%, 95%CI = 13–53%; *p* = 0.015) and tended toward increased diagnostic accuracy (non-obese: DOR = 14.44, 95%CI = 1.40–140.70; obese: DOR = 1.36, 95%CI = 0.52–3.59; *p* = 0.46) when compared to the obese cohort.

## 4. Discussion

ETE is an important parameter when considering patient prognosis. An abundance of literature suggests ETE to be a risk factor of poor prognosis, affecting both disease recurrence and patient survival [4,7,8,9,10,11]. Accordingly, accurate prediction of ETE preoperatively could influence surgical decision making and potentially improve patient outcomes. Our work found that US was of moderate diagnostic accuracy in predicting ETE. 

Patients with ETE are currently determined to have stage 3 cancer by the current American Thyroid Association (ATA) guidelines. Though prognosis is generally good in PTC patients, stage 3 cancer in these patients could have an impact on survival rate, especially if surgical resection is incomplete [20]. Resultantly, the use of a high-frequency linear transducer (i.e., 18 MHz) during US can improve diagnostic accuracy and assist in the patient care plan [2]. In our study, the overall specificity and sensitivity of US in detecting ETE were 64% and 43%, respectively. Accordingly, the use of adjunct imaging modalities such as CT or MRI may be advisable to improve preoperative ETE accuracy detection rates. Though our work found that no single sonographic feature could predict ETE with both high sensitivity and specificity, it could be the case that concurrent detection of multiple sonographic features could improve ETE predictive value. For example, Kamaya et al. found that the positive predictive value (PPV) of ETE detection was 32% when two or more sonographic features were present, 42% when three or more sonographic features were present, and 57% when all four sonographic features were present [2]. 

BMI has been suggested to influence US accuracy as excess adipose tissue adversely impacts the quality of the distance-dependent imaging modality [14,18]. A recent 2020 work reported that the sensitivity of US dropped significantly as BMI increased, determining hepatocellular carcinoma with 59% sensitivity in non-obese patients (BMI < 30) and only 19% sensitivity in obese patients (BMI ≥ 30) [21]. With respect to the thyroid, one study investigating US diagnostic testing accuracy found poorer lymph node metastasis detection accuracy (*p* = 0.025), but not extrathyroidal extension (*p* = 0.308), in obese (≥30 kg/m^2^) patients when compared with non-obese (<30 kg/m^2^) patients [19]. Our work found significantly decreased sensitivity in ETE detection by US in obese patients when compared with non-obese patients (*p* = 0.001). Importantly, the AUC for pooled sonographic features was significantly higher in non-obese patients compared with obese patients (*p* = 0.001). With respect to overall diagnostic performance, ETE detection by US was significantly higher in non-obese patients compared with obese patients (*p* = 0.03). This may explain why US assessment of ETE was reported with an accuracy of 83.3% (20/24 patients) in a Japanese-based study, where individuals are generally of lower weight class [22].

ETE assessment by US has been investigated previously in a handful of studies [2,4,23]. Unsurprisingly, Kwak et al. found that increased tumor contact with the adjacent capsule was associated with increased risk of ETE [23]. One study of 568 patients found that sonographic determination of tumor location (isthmus vs. non-isthmus), tumor size, and lymph node staging were each associated with ETE [4]. A different work of 62 patients found that the presence of capsular abutment was very sensitive (100%) in predicting ETE [2]. In our study, the sensitivity of capsular abutment in predicting ETE by US was 55%. Additionally, the authors reported that contour bulging was less sensitive (88%) than capsular abutment (100%) [2]. Although our values were considerably lower, which can be attributable to the likely higher BMI in our United States-based population, our results support this finding (34% versus 55% sensitivity, respectively). Finally, the authors found that loss of the echogenic capsule to be the best predictor of ETE (OR = 10.23, *p* = 0.34) [2], which corroborated our study findings. We found that, when considering all patients, loss of the echogenic capsule was the best predictor of ETE and possessed an impressive specificity of 88%. Importantly, loss of the echogenic capsule did not differ between the two BMI-based cohorts with respect to DOR (*p* = 0.51), specificity (*p* = 0.52), nor sensitivity (*p* = 0.09). Considering our work and the work of others [2], loss of the echogenic capsule could be considered the most important predictor of ETE when utilizing US. 

Finally, ultrasound can also be utilized as a non-invasive method of examining vocal cord function. A recent meta-analysis found that transcutaneous laryngeal ultrasound assessment of vocal cord function was concordant with laryngoscopy in 95.7% of patients [24]. Future studies are warranted to determine whether patient BMI may also influence transcutaneous laryngeal ultrasound assessment accuracy. 

## 5. Conclusions

Our work suggests that the diagnostic value of ETE determination by US is impaired in obese patients. Considering that loss of the echogenic capsule did not differ with respect to diagnostic performance, specificity, nor sensitivity between non-obese and obese patients, it could be considered the most important predictor of US-determined ETE.

## Figures and Tables

**Table 1:** Characteristics of study population. Data is presented as mean ± standard deviation and frequency with its corresponding percentage (%).

	All Patients (*n* = 204)	ETE Absent (*n* = 155)	ETE Present (*n* = 49)	*p*-Value
Age (years)	50.4 ± 15.1	50.4 ± 15.2	49.4 ± 15.8	0.71
Females	169 (83%)	144 (82%)	27 (93%)	0.14
BMI (kg/m^2^)	32.0 ± 8.9	32.0 ± 8.9	31.1 ± 8.4	0.24
Non-obese	42 (20.58%)	36 (17.64%)	6 (2.94%)	0.35
Obese	162 (79.41%)	126 (61.76%)	36 (17.64%)	
Mean tumor size (cm)	1.2 ± 1.3	1.2 ± 1.3	1.8 ± 1.4	0.04
Multifocality	84 (41.17%)	68 (33.33%)	16 (7.84%)	0.09

**Table 2:** Analysis of different sonographic features in assessing for extrathyroidal extension according to patient BMI. ETE: extrathyroidal extension. DOR: diagnostic odds ratio. PLR: positive likelihood ratio. NLR: negative likelihood ratio.

	Sonographic Feature	Non-Obese	Obese	*p*-Value
Sensitivity	ETE	0.83 (0.36–1.00)	0.30 (0.13–0.53)	0.015
	Capsular abutment	0.67 (0.22–0.96)	0.52 (0.31–0.73)	0.52
	Bulging of contour	0.50 (0.12–0.88)	0.30 (0.13–0.53)	0.37
	Loss of echogenic capsule	0.67 (0.22–0.96)	0.30 (0.13–0.53)	0.09
	Peripheral vascularity	0.83 (0.36–1.00)	0.39 (0.20–0.62)	0.05
	Pooled sensitivity	0.70 (0.51–0.85)	0.37 (0.28–0.46)	0.001
Specificity	ETE	0.74 (0.57–0.88)	0.76 (0.68–0.83)	0.81
	Capsular abutment	0.40 (0.24–0.58)	0.46 (0.37–0.54)	0.53
	Bulging of contour	0.71 (0.54–0.85)	0.64 (0.55–0.72)	0.07
	Loss of echogenic capsule	0.83 (0.66–0.93)	0.89 (0.83–0.94)	0.52
	Peripheral vascularity	0.31 (0.17–0.49)	0.48 (0.39–0.57)	0.08
	Pooled specificity	0.60 (0.52–0.67)	0.64 (0.61–0.68)	0.32
PLR	ETE	3.24 (1.66–6.32)	1.25 (0.63–2.48)	0.07
	Capsular abutment	1.11 (0.59–2.08)	0.96 (0.63–1.46)	0.74
	Bulging of contour	1.75 (0.67–4.55)	0.84 (0.43–1.61)	0.24
	Loss of echogenic capsule	3.89 (1.55–9.78)	2.84 (1.30–6.21)	0.68
	Peripheral vascularity	1.22 (0.80–1.85)	0.75 (0.44–1.28)	0.29
	Pooled PLR	1.85 (1.12–3.07)	1.09 (0.74–1.62)	0.13
NLR	ETE	0.22 (0.04–1.36)	0.92 (0.69–1.22)	0.029
	Capsular abutment	0.83 (0.25–2.77)	1.05 (0.66–1.66)	0.71
	Bulging of contour	0.70 (0.31–1.60)	1.09 (0.81–1.47)	0.56
	Loss of echogenic capsule	0.40 (0.13–1.26)	0.78 (0.59–1.03)	0.15
	Peripheral vascularity	0.53 (0.08–3.39)	1.27 (0.88–1.84)	<0.001
	Pooled NLR	0.57 (0.33–0.97)	0.98 (0.83–1.16)	0.29
DOR	ETE	14.44 (1.4–140.7)	1.36 (0.52–3.59)	0.46
	Capsular abutment	1.33 (0.21–8.29)	0.92 (0.38–2.22)	0.78
	Bulging of contour	2.50 (0.43–14.54)	0.76 (0.30–1.98)	0.38
	Loss of echogenic capsule	9.67 (1.43–65.38)	3.65 (1.29–10.29)	0.51
	Peripheral vascularity	2.29 (0.24–22.02)	0.59 (0.24–1.45)	0.54
	Pooled DOR	3.70 (1.53–8.94)	1.12 (0.62–2.03)	0.030

## Data Availability

Data are available per request from the corresponding author.
